# Subjective donor deferral as a tool for increased blood transfusion safety: A cross‐sectional observational study

**DOI:** 10.1002/hsr2.424

**Published:** 2021-10-14

**Authors:** Juliane Girão de Moura, Bruno Almeida Costa, Fabiana Aguiar Carneiro Silva, Francisco Vagnaldo Fechine, Ênio Simas Macedo, José Lucio Jorge Barbosa, Franklin José Candido Santos, Elizabeth de Francesco Daher, Luciana Maria de Barros Carlos, Denise Menezes Brunetta

**Affiliations:** ^1^ Department of Hematology Center of Hematology and Hemotherapy of Ceará (HEMOCE) Fortaleza Brazil; ^2^ Department of Medicine, Icahn School of Medicine at Mount Sinai Mount Sinai Morningside and Mount Sinai West New York New York USA; ^3^ Clinical Pharmacology Unit, Drug Research and Development Center (NPDM) Federal University of Ceará (UFC) Fortaleza Brazil; ^4^ Walter Cantidio Teaching Hospital (HUWC) Federal University of Ceará (UFC) Fortaleza Brazil

**Keywords:** blood donation testing, blood safety, donor health, donor recruitment, serologic testing, transfusion‐transmissible infections

## Abstract

**Objectives:**

This study aims at evaluating whether subjective donor deferral (SDD) has the potential for increasing blood transfusion safety.

**Background:**

Appropriate donor selection via clinical and serologic screening is necessary to prevent transfusion‐transmissible infections (TTIs). One additional strategy adopted by some Brazilian blood transfusion centers (BTCs) is the rejection of a donation by the pre‐donation interviewer based on subjective factors.

**Methods/Materials:**

We conducted a STROBE‐guided cross‐sectional study including 105 005 prospective donors who presented to our BTC between 1 January 2013, and 31 December 2015. Donors were evaluated for age, gender, education level, donation type and history, confidential unit exclusion, SDD, and results of serologic screening for TTIs.

**Results:**

Even after controlling for potential confounding variables, subjectively deferred donors were more likely to have at least one reactive serology in the standard screening (OR: 2.80; 95% CI: 2.13‐3.69; *P* < .001). They also had a higher risk for testing positive for syphilis (OR: 4.47; 95% CI: 3.05‐6.55; *P* < .001), hepatitis B (OR: 5.69; 95% CI: 2.48‐13.08; *P* < .001), and HIV (OR: 6.14; 95% CI: 3.22‐11.69; *P* < .001).

**Conclusions:**

Routine implementation of SDD in donor selection may be an effective additional measure to avoid TTIs, highlighting the importance of interviewer experience, perspicacity, and face‐to‐face contact with donors for blood safety assurance.

## 1 INTRODUCTION

In Brazil, many tools ensure the safety of hemotherapy. Initially, candidates for blood donation are evaluated with a brief epidemiological survey. Then, donor selection is performed through three major steps: a pre‐triage, in which weight, blood pressure, temperature, and heart rate are assessed; a hematological triage, in which hemoglobin and/or hematocrit levels are checked; and a pre‐donation interview with a qualified health professional. In the latter, the clinical and epidemiological history of the candidate is, as well as their actual health state and habits, reviewed.^1^

Subsequently, the donors' blood samples undergo a standard laboratory screening, where serologic and molecular biology tests are performed to identify potential transfusion‐transmissible infections (TTIs). Currently, Chagas disease, human immunodeficiency virus (HIV) I and II, human T‐lymphotropic virus (HTLV) I and II, syphilis, hepatitis B virus (HBV), and hepatitis C virus (HCV) infections are routinely evaluated.^2^

The Confidential Unit Exclusion (CUE) system is also recognized by Brazilian law. It allows donors to indicate privately that their blood donation may be unsafe for transfusion; as a result, blood collection proceeds as usual, but the bag collected is then discarded.^3^ Finally, an additional strategy is the subjective donor deferral (SDD), which means the possibility for discarding blood bags based on data perceived subjectively by the pre‐donation interviewer (eg, signs given by the prospective donor during the interview that may indicate he/she is failing with the truth or trying to hide important health information).^4^

Despite being empirically adopted by several Brazilian blood transfusion centers (BTCs), SDD is still an under‐researched tool that has not been formally validated in the previous literature. In this scenario, this study primarily aims at evaluating whether the SDD strategy has the potential to improve blood transfusion safety.

## 2 MATERIALS AND METHODS

### 2.1 Study design and participants

This is an observational, monocentric, cross‐sectional study including candidates for blood donation who presented to the Hematology and Hemotherapy Center of Ceará (HEMOCE) between 1 January 2013, and 31 December 2015. HEMOCE, located in Fortaleza, Brazil, is the primary supplier of blood and blood products for Ceará, a state with 8.8 million inhabitants in Northeast Brazil. Data were collected between January 2018 and March 2018 from the Blood Bank System (Sistema de Banco de Sangue; SBS) Web ISBT 128, the local database where all clinical and demographic information of donors is stored.

The prospective donors were evaluated according to the following parameters: gender (male or female), age (≤20 years, 21‐20 years, 31‐40 years, 41‐50 years, and >50 years), years of education (≤8 years, 9‐11 years, >11 years), donation history (first‐time donor or repeat donor), donation type (spontaneous or replacement), CUE option (exclusion or no exclusion), SDD (yes or no), and serologic test results.

**FIGURE 1:**
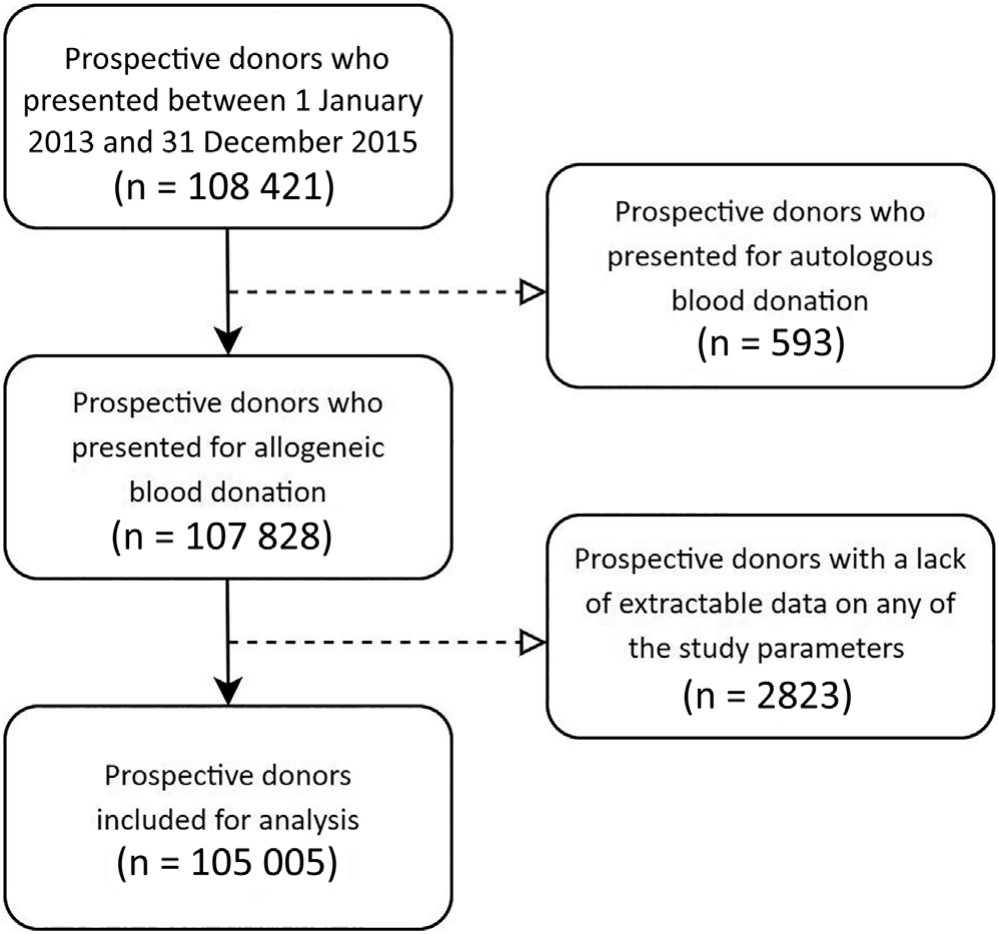
Flowchart of prospective blood donors included in the analysis after applying exclusion and inclusion criteria

The inclusion criteria were age ≥18 years and presentation for blood donation between 1 January 2013, and 31 December 2015. The exclusion criteria were intent for autologous blood donation and lack of Blood Bank System data on any of the parameters analyzed (eg, gender, age, years of education, donation history, CUE, SDD, or serologic test results). The flowchart of the study selection process is presented in Figure 1.

All blood samples were initially screened for the following: hepatitis B surface antigen (HBsAg) and anti‐HIV1/2 antibodies by Elecsys electrochemiluminescence immunoassay (Roche Diagnostics, Penzberg, Germany); anti‐hepatitis B core antigen (anti‐HBc) and anti‐HTLV1/2 antibodies by ARCHITECT chemiluminescence immunoassays (Abbott Laboratories, Abbott Park, Illinois, USA); and anti‐HCV antibodies by VITROS chemiluminescence immunoassay (Ortho Clinical Diagnostics, Raritan, New Jersey, USA). In addition, Oxoid venereal disease research laboratory (VDRL) test kit (Thermo Fisher Scientific, Basingstoke, UK) was used for syphilis screening. For HIV nucleic acid testing (NAT), the Superscript III Platinum One‐Step Quantitative RT‐PCR kit (Thermo Fisher Scientific, Life Technologies, Carlsbad, CA) was used. Samples with positive results were retested by the same method and using the same equipment. Two positive tests defined a sample as reactive for a specific infectious agent. Individuals with at least one reactive serology after standard laboratory screening were considered as having “positive general serologic screening.” Individuals with no reactive serology after standard laboratory screening were considered as having “negative general serologic screening.” Despite being routinely performed in HEMOCE, serology for Chagas disease was not considered in this analysis due to the lack of association between risky sexual behavior and active infection.

During the analysis, we evaluated whether SDD was established or not for each of the study participants. SDD can be defined as an additional strategy that some BTCs use in the attempt to reduce transfusion of blood from individuals in the window period for TTIs. It consists of discard of the collected blood based on data that were not reported by the prospective donor during clinical triage but instead perceived subjectively by the pre‐donation interviewer. The decision for SDD can be performed concomitantly, before, or after the clinical triage. Although serologic testing is still performed in blood samples of subjectively deferred individuals, the blood bag collected is always discarded, even if initial serologic/molecular testing is negative.

Finally, all first‐time donors submitted to SDD and with negative serologic/molecular testing were identified and, in those who returned for a second donation, the presence or absence of seroconversion for a sexually transmitted infection (STI) was evaluated.

The local Ethics Committee approved the study protocol (approval number 86154618.7.0000.8152) and provided exemption from the need for informed consent, as this study involved no risks to subjects, could not be carried out practicably without the waiver, and analyzed data already available in the Blood Bank System. In addition, the waiver would not adversely affect the rights or welfare of the subjects, who would still receive standard of care and have the privacy of their personal/health information guaranteed. Of note, we have conducted the present research according to the Strengthening the Reporting of Observational Studies in Epidemiology (STROBE) guidelines for cross‐sectional studies.

### 2.2 Statistical analysis

Descriptive statistics of categorical variables comprised the calculation of absolute frequency (n) and relative frequency (%). The association between sociodemographic factors and clinical triage information (explanatory variables) and the primary outcome variable (ie, the result of the general serologic screening of blood donors) was evaluated using univariate logistic regression analysis. The strength of this association was measured by determining the crude odds ratio (OR), as well as the accuracy (95% confidence interval; 95% CI) and significance (Wald test) of the estimate. In order to evaluate each disease individually, the same approach was repeated with HIV, HBV, and syphilis serologic results as separate outcome variables.

For each of the outcomes evaluated (ie, results of the general serologic screening and serologic screening for HIV, HBV, and syphilis), the explanatory variables associated with it at a significance level of 10% (*P* < .10) were selected to integrate the model of multivariate logistic regression. The backward stepwise regression method was used to identify factors independently associated with each outcome. Such analysis allowed the determination of adjusted OR, as well as accuracy (95% CI) and significance (Wald test) of the estimate. In all analyses, two‐tailed tests were used, and a significance level of .05 was established. IBM SPSS Statistics software version 23.0 (IBM Corp., Armonk, NY, USA, 2015) was used to perform all statistical procedures.

## 3 RESULTS

### 3.1 Demographic, serologic, and clinical triage data

Among the 105 005 prospective donors included for analysis, 665 (0.63%) confidentially excluded their blood and 1115 (1.06%) were subjectively deferred by the interviewer. Of note, 96.5% of the subjectively deferred individuals reported their blood to be safe (ie, only 39 of them performed CUE). Further donor selection data are described in Table 1.

**TABLE 1:** Sociodemographic characteristics and information on clinical and serologic screening of blood donors from a blood center

Characteristic	Absolute frequencies	Relative frequencies (%)
Gender		
Male	64 328	61.26
Female	40 677	38.74
Age range		
>50 y	7371	7.02
41–50 y	16 188	15.42
31–40 y	30 412	28.96
21–30 y	39 821	37.92
≤20 y	11 213	10.68
Years of study		
≤8 y	14 087	13.42
9–11 y	61 631	58.69
>11 y	29 287	27.89
Donation history		
First‐time donor	65 333	62.22
Repeat donor	39 672	37.78
Donation type		
Replacement	17 068	16.25
Spontaneous	87 937	83.75
Confidential unit exclusion		
Self‐exclusion	665	0.63
No self‐exclusion	104 340	99.37
Subjective donor deferral		
Yes	1115	1.06
No	103 890	98.94
Anti‐HIV1/2		
Reactive	152	0.14
Non‐reactive	104 853	99.86
VDRL		
Reactive	641	0.61
Non‐reactive	104 364	99.39
Anti‐HTLV1/2		
Reactive	130	0.12
Non‐reactive	104 875	99.88
HBsAg		
Reactive	110	0.10
Non‐reactive	104 895	99.90
Anti‐HBc		
Reactive	944	0.90
Non‐reactive	104 061	99.10
Anti‐HCV		
Reactive	271	0.26
Non‐reactive	104 734	99.74
General serologic screening		
≥1 reactive serology	2108	2.01
No reactive serology	102 897	97.99

*Note*: Data expressed as absolute (n) and relative (%) frequency.

### 3.2 General serologic screening

Univariate analysis of the association between sociodemographic/clinical triage data and a positive general serologic screening is shown in Table 2. Increasing age, decreasing education, replacement donations, and repeat donations were significantly associated with ≥1 reactive serology for the pathogens researched. Option for CUE (OR: 1.68; 95% CI: 1.09‐2.58; *P* = .018) and occurrence of SDD (OR: 2.68; 95% CI: 2.04‐3.50; *P* < .001) were also risk factors for ≥1 reactive serology. All those variables were included in a multivariate regression model, shown in Table 3.

**TABLE 2:** Univariate logistic regression analysis of the association between different donor characteristics and general serologic screening results with the determination of crude odds ratio as well as accuracy (95% confidence interval) and significance (Wald test) of the estimate

Characteristic	General serologic screening				Crude OR	95% CI	Significance
	≥1 reactive serology		No reactive serology				
	n	%	n	%			
Gender							
Male	1319	2.05	63 009	97.95	1.06	0.97‐1.16	*P* = .213
Female	789	1.94	39 888	98.06	1		
Age range							
>50 y	239	3.24	7132	96.76	**2.19**	1.79‐2.67	***P* < .001**
41–50 y	388	2.40	15 800	97.60	**1.60**	1.34‐1.93	***P* < .001**
31‐40 y	611	2.01	29 801	97.99	**1.34**	1.13‐1.59	***P* = .001**
21‐30 y	701	1.76	39 120	98.24	1.17	0.99‐1.39	*P* = .068
≤20 y	169	1.51	11 044	98.49	1		
Years of study							
≤8 y	445	3.16	13 642	96.84	**2.39**	2.09‐2.74	***P* < .001**
9–11 y	1269	2.06	60 362	97.94	**1.54**	1.38‐1.73	***P* < .001**
>11 y	394	1.35	28 893	98.65	1		
Donation history							
First‐time donor	527	0.81	64 806	99.19	**0.20**	0.18‐0.22	***P* < .001**
Repeat donor	1581	3.99	38 091	96.01	1		
Donation type							
Replacement	402	2.36	16 666	97.64	**1.22**	1.09‐1.36	***P* < .001**
Spontaneous	1706	1.94	86 231	98.06	1		
Confidential unit exclusion							
Self‐exclusion	22	3.31	643	96.69	**1.68**	1.09–2.58	***P* = .018**
No self‐exclusion	2086	2.00	102 254	98.00	1		
Subjective donor deferral							
Yes	57	5.11	1058	94.89	**2.68**	2.04–3.50	***P* < .001**
No	2051	1.97	101 839	98.03	1		

*Note*: In the general serologic screening, the result was considered reactive when the serologic testing was positive for at least one of the pathogens researched. Data expressed as absolute (n) and relative (%) frequency. In this table, all statistically significant crude odds ratio values (*P* ≤ 0.05) and their respective significance levels were formatted in bold.

Abbreviations: OR, odds ratio; 95% CI, 95% confidence interval of the crude odds ratio.

**TABLE 3:** Determination of the factors associated with a reactive general serologic screening of blood donors from a blood center, after controlling for possible confounding variables

Characteristic	Univariate analysis	Multivariate analysis		Significance
	Crude OR	Adjusted OR	95% CI	
Age range				
>50 y	2.19	**4.00**	3.25‐4.92	***P* < .001**
41–50 y	1.60	**2.90**	2.40‐3.50	***P* < .001**
31‐40 y	1.34	**2.43**	2.04‐2.90	***P* < .001**
21–30 y	1.17	**1.75**	1.48‐2.08	***P* < .001**
≤20 y	1	1		
Years of study				
≤8 y	2.39	**1.67**	1.45‐1.93	***P* < .001**
9–11 y	1.54	**1.50**	1.34‐1.68	***P* < .001**
>11 y	1	1		
Donation history				
First‐time donor	0.20	**0.17**	0.15‐0.19	***P* < .001**
Repeat donor	1	1		
Donation type				
Replacement	1.22	0.91	0.82‐1.02	*P* = .111
Spontaneous	1	1		
Confidential unit exclusion				
Self‐exclusion	1.68	1.32	0.86–2.04	*P* = .210
No self‐exclusion	1	1		
Subjective donor deferral				
Yes	2.68	**2.80**	2.13–3.69	***P* < .001**
No	1	1		

*Note*: Multivariate logistic regression analysis was used to determine the adjusted odds ratio, as well as the 95% confidence interval and the significance of the estimate. In this table, all statistically significant adjusted odds ratio values (*P* ≤ 0.05) and their respective significance levels were formatted in bold.

Abbreviations: OR, odds ratio; 95% CI, 95% confidence interval of the adjusted odds ratio.

After multivariate regression analysis, increasing age, decreasing education, and repeat donations remained significantly associated with higher odds of a positive general serologic screening. After adjusting for the contribution of other factors, individuals submitted to SDD were found to be more likely to have ≥1 reactive serology than the ones who were not deferred by interviewers (OR: 2.80; 95% CI: 2.13‐3.69; *P* < .001). On the other hand, option for CUE was no longer associated with a positive general serologic screening (OR: 1.32; 95% CI: 0.86‐2.04; *P* = .210).

### 3.3 HIV serologic testing

After univariate analysis, age >50 years (OR: 0.23; 95% CI: 0.07‐0.77; *P* = .017) and first‐time donation (OR: 0.40; 95% CI: 0.29‐0.55; *P* < .001) were suggested as protective factors for reactive HIV serology. In contrast, SDD was suggested as a risk factor (OR: 6.61; 95% CI: 3.47‐12.59; *P* < .001). Due to *P* values >.10, the other variables were not included in the multivariate analysis.

When controlling for possible confounding variables was performed, individuals submitted to SDD were more likely to have positive HIV serology than those who were not (OR: 6.14; 95% CI: 3.22‐11.69; *P* < .001). Although the OR for a first‐time donation remained the same (OR: 0.40; 95% CI: 0.29‐0.56; *P* < .001), age >50 years was no longer associated with a positive HIV serology (OR: 0.35; 95% CI: 0.10‐1.17; *P* = .087).

### 3.4 Syphilis serologic testing

Univariate analysis suggested replacement donation (OR: 1.28; 95% CI: 1.05‐1.55; *P* = .015), option for CUE (OR: 2.25; 95% CI: 1.16‐4.37; *P* = .016), SDD (OR: 4.51; 95% CI: 3.09‐6.57; *P* < .001), age >50 years (OR: 1.55; 95% CI: 1.07‐2.26; *P* = .021), and either 9‐11 years study (OR: 1.82; 95% CI: 1.47‐2.27; *P* < .001) or <8 years of study (OR: 2.93; 95% CI: 2.27‐3.77; *P* < .001) as risk factors for a positive syphilis screening.

When multivariate analysis was performed, replacement donation (OR: 1.02; 95% CI: 0.83‐1.24; *P* = .880) and option for CUE (OR: 1.71; 95% CI: 0.87‐3.33; *P* = .117) were no longer significantly associated with a reactive VDRL. However, donors aged 21‐30 years (OR: 1.86; 95% CI: 1.39‐2.50; *P* < .001) or >50 years (OR: 2.32; 95% CI: 1.57‐3.41; *P* < .001) were more likely to have a reactive VDRL than those aged ≤20 years. In addition, individuals with length of schooling ≤8 years (OR: 2.49; 95% CI: 1.91‐3.24; *P* < .001) or between 9 and 11 years (OR: 1.83; 95% CI: 1.47‐2.27; *P* < .001) were more likely to have a reactive VDRL than those with >11 years of education. Conversely, a first‐time donation seemed to be a protective factor (OR: 0.27; 95% CI: 0.23‐0.32; *P* < .001). Most importantly, subjectively deferred donors had significantly higher odds of having a reactive VDRL (OR: 4.47; 95% CI: 3.05‐6.55; *P* < .001).

### 3.5 Hepatitis B serologic testing

After univariate analysis, first‐time donation (OR: 0.15; 95% CI: 0.10‐0.24; *P* < .001), length of schooling ≤8 years (OR: 2.68; 95% CI: 1.51‐4.74; *P* = .001), age of 31‐40 years (OR: 2.11; 95% CI: 0.94‐4.71; *P* = .069), SDD (OR: 5.40; 95% CI: 2.37‐12.32; *P* < .001), and first‐time donation (OR: 0.15; 95% CI: 0.10‐0.24; *P* < .001) met criteria to integrate the multivariate logistic regression model.

After controlling for possible confounding variables, age >50 years (OR: 4.51; 95% CI: 1.71‐11.93; *P* = .002), between 41 and 50 years (OR: 4.02; 95% CI: 1.69‐9.58; *P* = .002), or between 31 and 40 years (OR: 4.14; 95% CI: 1.84‐9.30; *P* = .001) was shown to be a risk factor for a reactive HBsAg. First‐time donation remained a protective factor for positive HBV screening (OR: 0.13; 95% CI: 0.08‐0.20; *P* < .001). Finally, SDD was significantly associated with a reactive HBsAg (OR: 5.69; 95% CI: 2.48‐13.08; *P* < .001).

### 3.6 Donor seroconversion

During the study period, the frequency of return of the subjectively deferred donors to the BTC was also evaluated. Only 60 (5.4%) of the 1115 individuals submitted to SDD returned for a second donation. Of those, two tested positive for HIV, four for syphilis, and one for anti‐HBC, corresponding to 11.7% of seroconversion.

## 4 DISCUSSION

SDD was demonstrated to be a helpful donor triage tool in this cross‐sectional study, remaining a statistically significant risk factor for all the analyzed outcomes even when other variables' influence was controlled. Subjectively deferred donors were 2.80 times more likely to have at least one positive screening test, 4.47 times more likely to have positive syphilis serology, 5.69 times more likely to have positive HBV serology, and 6.14 times more likely to have positive HIV serology. As the rates of positive infectious disease markers can correlate with the incidence of window‐period infections,^5^ the data herein presented suggest that the SDD strategy has the potential to improve blood transfusion safety.

Although serologic testing is still performed in subjectively deferred blood, the donation collected is discarded, even if all screening tests are negative. While O'Brien et al reported the residual risk of HIV to be 1 per 8 million donations,^6^ when we evaluated the 60 subjectively deferred donors who returned to the BTC for a second donation, HIV seroconversion was seen in two of them. Hypothesizing that those individuals were in the window period in their first donation, SDD could have prevented transfusion‐transmitted HIV infection in six recipients (as one bag of packed red blood cells, one bag of platelet concentrate, and one bag of fresh frozen plasma derive from each donation). Therefore, individuals in the window period for an STI may potentially be detected through this tool, decreasing the risk for TTIs in the recipient.

Identifying the local pattern of donor deferral provides critical areas to focus on policy formulation for donor selection.^7^ Previous studies have shown older patients to have increased odds for anti‐HBc, HBsAg, anti‐HCV, and anti‐HTLV1/2 reactivity.^8^, ^9^, ^10^ Accordingly, the present study showed increasing age to be a risk factor for reactive HBsAg and positivity in at least one of the serologic tests performed. Although syphilis incidence is higher in the younger patient population,^11^ our analysis suggested that older donors are more likely to have a positive VDRL, in agreement with Vera et al and Jaques et al, who also analyzed VDRL results in populations of volunteer blood donors.^12^, ^13^ Our results indicated that no specific age range is associated with higher odds of HIV infection, contrasting with previous studies suggesting that younger age is a risk factor.^14^, ^15^ The current rapid increase in the prevalence of HIV‐infected people aged 50 years or older may have contributed to such an outcome.^16^ Lower education level was associated with a higher risk for positive general serological screening and positive syphilis screening, in consonance with previous literature data.^17^ However, a similar association was not found for a positive screening for HIV or HBV. Similar to gender, donation type had no significant association with any of the outcomes described, which contrasted with the results of Jaques et al, who showed replacement donations were more likely to be HBV‐ or HIV‐infected than spontaneous donations.^13^

In all the analyses conducted, subjectively deferred donors had higher odds of a positive screening test than donors who confidentially excluded their blood. Of note, when multivariate regression models were performed, opting for CUE was not a statistically significant risk factor for positive general serologic screening, HIV serology, HBV serology, or syphilis serology. Although other studies found an association of CUE with a higher prevalence of reactive markers for HIV, HBV, and syphilis, they also showed CUE to have a limited effect on reducing TTIs secondary to window‐period donations.^5^, ^18^, ^19^ Also, CUE may be a confusing method, with donors often opting for CUE by mistake,^20^ and rarely prevents the transfusion of units from donors with undisclosed high‐risk deferrable behavior.^21^ Besides, while SDD highlights the importance of the healthcare worker's experience and perspicacity for blood safety assurance, CUE use may reduce the perceived responsibility of staff on eliciting a history of high‐risk behavior.^22^ Finally, SDD represents no additional costs for BTCs.^4^ With that in mind, the SDD strategy, which is only used in some Brazilian BTCs, could be a better tool for blood safety improvement than CUE, which is already implemented in many BTCs worldwide.

The decision for SDD can derive from concrete information or suspected situations that could compromise transfusion safety, with such data not being reported by the prospective donor during the pre‐donation interview but instead perceived independently by the interviewer. In a recent qualitative analysis, the primary reasons reported by BTC staff interviewers as determinants for SDD were suspicion of lack of truth or omission in the donor's speech (eg, answers unrelated to the question, attempts to manipulate the answers, apparent insecurity in the answers) and donor's behavior during or after screening (eg, disrespectful, disinterested, disoriented, excessively nervous). Other motivations for SDD were denial of registered deferral reasons from previous donation attempts, excessive interest in the post‐donation serologic results, donor's sexual partner blockade in the BTC system due to a reactive serology, and collateral information from a third‐party acquainted with the donor.^4^ However, due to its subjective nature, the SDD tool is difficult to standardize, creating the need for further studies to define the criteria used by the interviewers more clearly.

Despite the large sample size and robust statistics, the present study has several limitations. Due to its strong regional focus, the results may be too population‐specific. Thus, a multicentric study is needed to assess further the potential of SDD for preventing TTIs. In addition, direct interference with the amount of collected bags used for transfusion may be a major drawback. Considering that CUE has been shown to cause a low but consistent discard rate of safe units,^21^ it is essential to analyze whether SDD can cause a similar effect in future studies. Finally, there are some ethical standpoints to consider: Does the method reflects the staff's inability to develop empathy and confidentiality with the donor? In order to reduce bias, should more than one healthcare professional decide if the donor's blood ought to be discarded? As the current SDD policy does not demand informing donors of the interviewers' decision, is it ethical to subject the individuals to the illusion and risks of donating if the blood will not be transfused?

Nevertheless, in an era in which computerized questionnaires and computer‐assisted self‐interviews are increasingly discussed and adopted as substitutes for the traditional pre‐donation interviews,^23^, ^24^ the present study highlights the importance of the healthcare worker experience and face‐to‐face contact with the prospective donor for assurance of blood safety, especially in low‐income regions where associated costs and technical complexity may limit the use of modern molecular testing methods for the prevention of TTIs secondary to window‐period donations.^25^

## 5 CONCLUSION

The implementation of SDD as an adjunct tool for clinical triage of blood donors may be an effective way to increase the detection of individuals at risk of transmitting infectious diseases, such as HIV, HBV, and syphilis. The possibility of detecting donors in the window period for the cited infections may reduce TTIs in the recipients. However, further studies on SDD are needed to confirm this assertion.

## FUNDING

This research did not receive any specific grant from funding agencies in the public, commercial, or not‐for‐profit sectors.

## CONFLICT OF INTEREST

The authors declare that they have no conflicting interests.

## AUTHOR CONTRIBUTIONS

Conceptualization: Juliane Girão de Moura, Fabiana Aguiar Carneiro Silva, Denise Menezes Brunetta, Luciana Maria de Barros Carlos, Elizabeth de Francesco Daher

Data Curation: Juliane Girão de Moura, Fabiana Aguiar Carneiro Silva, Bruno Almeida Costa, Francisco Vagnaldo Fechine, Denise Menezes Brunetta

Formal Analysis: Bruno Almeida Costa, Francisco Vagnaldo Fechine, Ênio Simas Macedo

Methodology: Denise Menezes Brunetta, José Lucio Jorge Barbosa, Franklin José Candido Santos

Supervision: Elizabeth de Francesco Daher, Luciana Maria de Barros Carlos, Denise Menezes Brunetta

Writing – Original Draft Preparation: Juliane Girão de Moura, Bruno Almeida Costa, Francisco Vagnaldo Fechine, Ênio Simas Macedo, Fabiana Aguiar Carneiro Silva, Denise Menezes Brunetta

Writing – Review & Editing: José Lucio Jorge Barbosa, Franklin José Candido Santos, Elizabeth de Francesco Daher, Luciana Maria de Barros Carlos, Bruno Almeida Costa

All authors have read and approved the final manuscript version of the manuscript.

The corresponding author had full access to all of the data in this study and takes complete responsibility for the integrity of the data and the accuracy of the data analysis.

## TRANSPARENCY STATEMENT

The manuscript is an honest, accurate, and transparent account of the study being reported, and no important aspects of the study have been omitted.

## Data Availability

The authors confirm that the data supporting the findings of this study are available within the article.
